# Gut microbiota composition correlates with PBMC microRNA expression following maximal exercise testing in endurance athletes

**DOI:** 10.3389/frmbi.2026.1734737

**Published:** 2026-04-01

**Authors:** Guy Shalmon, Guy Shapira, Rawan Ibrahim, Ifat Israel-Elgali, Meitar Grad, Rani Shlayem, Ilan Youngster, Mickey Scheinowitz, Noam Shomron

**Affiliations:** 1 Sylvan Adams Sports Institute, School of Public Health, Gray Faculty of Medical and Health Sciences, Tel Aviv University, Tel Aviv, Israel; 2 Gray Faculty of Medical and Health Sciences, Tel Aviv University, Tel Aviv, Israel; 3 Edmond J Safra Center for Bioinformatics, Tel Aviv University, Tel Aviv, Israel; 4 Department of Biomedical Engineering, Faculty of Engineering, Tel Aviv University, Tel Aviv, Israel; 5 Pediatric Infectious Diseases Unit, The Center for Microbiome Research, Shamir Medical Center, Tel Aviv, Israel

**Keywords:** athletes, cyclists, exercise, gut microbiota, microbiome, microRNAs, PBMC, runners

## Abstract

**Introduction:**

MicroRNAs (miRNAs) are key post-transcriptional regulators that also take part in immune responses and recovery processes following exercise. While both gut microbiota composition and peripheral blood mononuclear cell (PBMC)-derived miRNAs are known to be influenced by endurance training, potential correlations between these two systems in athletes remain largely unexplored.

**Objective:**

This study aimed to investigate correlations between gut microbiota composition and PBMC miRNA expression following a maximal exercise stress test in endurance athletes.

**Methods:**

Fifty-eight participants (22 runners, 18 cyclists, and 18 controls) underwent maximal exercise testing, with blood samples collected pre- and post-maximal exercise stress test for small RNA sequencing of PBMCs. Baseline fecal samples were analyzed via 16S rRNA gene sequencing to characterize gut microbiota. Expression data of PBMC miRNAs and microbial taxonomic profiles were integrated to assess potential correlations.

**Results:**

Thirteen significant correlations (|r| = 0.41–0.51, p < 0.005) were identified between gut bacterial taxa known to produce short-chain fatty acids (SCFAs)—including *Veillonella, Blautia, Coprococcus, Butyrivibrio, Propionibacterium*, and *Parabacteroides*—and the expression of PBMC miRNAs following a maximal exercise test. The significantly expressed PBMC microRNAs included hsa-miR-545-3p, hsa-miR-126-3p, hsa-miR-1292-3p, hsa-miR-6805-5p, hsa-miR-3668, hsa-miR-196b-5p, hsa-miR-602, hsa-miR-324-5p, and hsa-miR-365a-3p, some of which are known to modulate inflammatory pathways and immune cell signaling.

**Conclusion:**

This is the first study demonstrating an association between resting gut microbiota composition and PBMC miRNA expression following maximal exercise stress test in endurance athletes. These findings raise the possibility of a complex association between gut microbial composition and PBMC miRNA expression in response to exercise. While causality cannot be inferred, the observed correlations suggest a candidate microbiota–miRNA that warrants further investigation in the context of exercise-induced immune regulation and recovery in athletes.

## 1 Introduction

MicroRNAs (also miRNAs) are short non-coding RNA molecules, typically 18–25 nucleotides in length, that regulate gene expression post-transcriptionally, primarily by binding to complementary sequences in target mRNAs, leading to translational repression or mRNA degradation (Shomron and Levy, 2009). These molecules are key regulators of numerous physiological processes, including immune responses, inflammation, metabolism, and cellular differentiation (O’Connell et al., 2012). Alterations in microRNA expression have been linked to a variety of pathophysiological conditions, and emerging evidence suggests that they also play critical roles in mediating the molecular adaptations induced by physical activity and endurance training (Fernandez-Sanjurjo et al., 2018).

Peripheral blood mononuclear cells (PBMCs), which include lymphocytes (T cells, B cells, and NK cells) and monocytes, serve as a readily accessible source of immune cells for profiling molecular responses to exercise (Theall et al., 2021). PBMCs are often used to study systemic immunological adaptations because they circulate throughout the body and respond to both acute and chronic physiological stimuli, including exercise-induced stress (Da Rosa et al., 2023). Given that exercise induces changes in PBMC microRNA expression, many of which are associated with inflammatory pathways, these profiles may offer valuable insights into immune modulation and recovery processes in active individuals and athletes (Radom-Aizik et al., 2012). Moreover, exercise-induced microRNA changes may contribute to broader systemic adaptations, offering additional insight into processes such as immune regulation and physiological recovery (Meurer et al., 2016).

In parallel, a growing body of research highlights the importance of gut microbiota in modulating host immunity, metabolism, and exercise performance (O’Brien et al., 2022; Devries and O’Neill, 2023; Patel et al., 2024). Endurance athletes typically exhibit a more alpha-diverse gut microbiota than sedentary individuals, potentially contributing to improved energy metabolism, anti-inflammatory responses, and recovery processes (Varghese et al., 2024), a finding that was also confirmed in our previous study (Shalmon et al., 2024), which demonstrated significantly higher gut microbial alpha-diversity in competitive non-professional female and male runners compared to sedentary controls. These changes in gut microbial composition are thought to result from long-term dietary patterns, high-volume training, and repeated exposure to physiological stressors (Mohr et al., 2020).

Studies from recent years have revealed significant correlations between gut microbiota composition and host microRNA expression (Rizk and Tüzmen, 2020; Sonoyama and Ohsaka, 2023). While the underlying mechanisms remain under investigation, emerging evidence suggests a potentially bidirectional interaction, wherein microbial metabolites may influence microRNA profiles, and host-derived microRNAs may, in turn, affect microbial communities (Rizk and Tüzmen, 2020; Sonoyama and Ohsaka, 2023). However, to date, these studies have been limited to clinical populations and have primarily focused on pathological conditions such as inflammatory bowel disease, cancer, and metabolic disorders. No studies have examined such interactions in the context of exercise or athletic performance. Direct correlations between gut bacterial taxa and microRNAs in athletes remain largely unexplored, particularly with regard to the relationship between PBMC microRNA expression and gut microbiota composition. Given the temporal and physiological distinction between gut microbiota stability at rest and the dynamic nature of PBMC microRNA expression in response to exercise, such correlations may reflect how baseline microbial profiles are associated with the host’s immune-related transcriptional responses to physical stress. Although gut bacteria do not produce microRNAs, they can modulate host gene expression through the production of metabolites and interactions with the immune system. Investigating these associations may therefore provide novel insights into microbiota–host communication pathways relevant to recovery, adaptation processes, and overall athletic performance.

The present study addresses this knowledge gap by investigating correlations between gut microbiota composition and PBMC microRNA expression following a maximal exercise test in endurance-trained athletes, including both female and male runners and cyclists.

By integrating microbiome mapping with Small RNA transcriptomics profiling of PBMCs, this study aims to identify specific microbial changes that might be associated also with microRNA expression, which may come together with a specific immune regulation, performance adaptation, and post-exercise recovery in athletes.

## 2 Materials and methods

### 2.1 Study design and participants

Endurance athletes running and cycling were recruited from competitive sports clubs, while control group participants were selected from the general population. A total of 58 individuals (31 males and 27 females) were enrolled in the study. This cohort comprised 18 cyclists (9 males), with a mean age of 46 ± 7 years; 22 runners (13 males), with a mean age of 43 ± 6.5 years; and 18 control participants (9 males), with a mean age of 41 ± 7.4 years. The athletes were amateur yet competitively active, engaging in rigorous training schedules and participating in domestic-level events. Cyclists were characterized as competitive endurance athletes who cycled no less than 120 kilometers per week, whereas runners were defined as those who ran a minimum of 50 kilometers weekly. In contrast, individuals in the control group engaged in low-intensity physical activity, with a weekly running distance of under 5 kilometers. Participants completed an online questionnaire providing detailed information regarding their weekly training routines. This included the frequency of training sessions per week, the average duration of each session, the relative intensity (measured as a percentage of maximal heart rate), and dietary habits—encompassing food types, portion sizes, and meal frequency. These data were used to evaluate their nutritional patterns, which are known to potentially affect gut microbiota composition.

Each participant underwent a maximal aerobic capacity test (VO2max), during which venous blood samples were collected immediately before and after exercise to evaluate the expression levels of microRNAs in peripheral blood mononuclear cells (PBMCs). In addition, a fecal sample was obtained from each participant for gut microbiota composition analysis.

Based on this assessment, only individuals whose diets consistently included meat, fish, fruits, vegetables, and grains in comparable proportions throughout the week were included. Participants exhibiting marked deviations in dietary intake were excluded to reduce inter-individual variability. All participants identified as Ashkenazi Jews with ancestral origins in Europe and North America. This homogeneity in ethnic background was intended to minimize cultural dietary differences that might confound microbiota-related outcomes. To further standardize the cohort, only omnivores were selected for inclusion. Individuals who had used nutritional supplements (including probiotics, prebiotics, multivitamins, or antacids such as beta-alanine or sodium bicarbonate) or had been treated with antibiotics within three months before the study were excluded.

All participants received a detailed explanation of the study protocol and provided written informed consent. The study protocol was reviewed and approved by the Ethics Committee of Tel Aviv University, Israel (Approval No. 0003766-1). Signed consent forms are securely stored by the principal investigator at Tel Aviv University.

### 2.2 Exercise test

Participants performed a maximal effort exercise test to simulate a competitive-like physiological load. While VO2max was determined during the test using a COSMED Quark metabolic cart (COSMED S.r.l., Rome, Italy) (Shephard, 1984; Evans et al., 2015), the primary aim was to induce a full-body, volitional exhaustion response under sport-specific conditions. Cyclists completed a ramp protocol on a stationary SRM ergometer, starting at 80 watts with increments of 20 watts per minute, maintaining a cadence of 80 rpm until volitional fatigue (typically around 60 rpm). Runners and control participants performed a treadmill running test (H/P Cosmos), starting at a speed equivalent to 50% of their running economy, with a one-kph increase every minute until exhaustion. Control subjects followed the same running protocol as runners, given that their habitual activity involved walking or light jogging rather than cycling.

### 2.3 Gut microbiome analysis

#### 2.3.1 Stool sample collection

Participants were given a sterile Stomacher^®^ bag (Seward Ltd., Worthing, West Sussex, UK) for fecal sample collection. They were given clear and consistent instructions on collecting the stool sample without compromising the quality of the sample. Upon receipt, the samples were aliquoted into sterile collection tubes and promptly stored at −80 °C within four hours of collection to preserve sample integrity for subsequent analyses. All stool processing procedures were conducted under strict anaerobic conditions to maintain the viability of obligate anaerobic microbiota.

#### 2.3.2 DNA extraction

Bacterial DNA was isolated from fecal samples using a combination of chemical lysis and mechanical disruption. Each sample (200 µL) was transferred into Type C bead-beating tubes (GeneAid, Taipei, Taiwan), to which 270 µL of GT lysis buffer and 30 µL of proteinase K (both from the MagCore Genomic DNA Tissue Kit, RBC Bioscience, Taipei, Taiwan) were added. Mechanical cell lysis was carried out by bead beating for 2 minutes using a BioSpec homogenizer (BioSpec Products, Bartlesville, OK, USA). Following homogenization, the samples were incubated at 60 °C for 2 hours to facilitate enzymatic digestion. Subsequent DNA purification was performed using the MagCore automated extraction system (RBC Bioscience, Taipei, Taiwan), following the manufacturer’s protocol for the MagCore Genomic DNA Tissue Kit cartridges.

#### 2.3.3 PCR

DNA concentrations were determined using a NanoDrop spectrophotometer (Thermo Fisher Scientific, Waltham, MA, USA), and approximately 20 ng of genomic DNA from each sample served as the template for the initial PCR amplification. The reaction was carried out in a 25 µL volume using the Hot Start Ready Mix (PCR Biosystems Ltd., London, UK) along with custom primers targeting the V4 region of the 16S rRNA gene, as specified by the Earth Microbiome Project (2023), which includes CS1/CS2 adaptor sequences. The first PCR was run for 25 cycles. Subsequently, 2 µL of the PCR1 product—containing the CS1/CS2 adaptors—was subjected to a second round of amplification using the Fluidigm Access Array Barcode Library. This step was conducted according to the manufacturer’s guidelines, with 2 µL of barcode added per 10 µL reaction volume, and 10 additional PCR cycles were performed (Fluidigm Corporation, 2019). PCR products were purified using Pure Beads (Roche Sequencing Solutions, Inc., Wilmington, DE, USA) at a bead-to-sample ratio of 0.65×. DNA quantification was performed using the Qubit dsDNA High Sensitivity Assay (DeNovix Inc., Wilmington, DE, USA), and fragment size distribution and integrity were assessed using the Agilent TapeStation system with DNA ScreenTape and associated reagents (Agilent Technologies Inc., Santa Clara, CA, USA).

#### 2.3.4 16S rRNA gene sequencing

Sequencing was performed using the Illumina MiSeq platform (Illumina Inc., San Diego, CA, USA), employing the MiSeq Reagent Kit v2 (500-cycle, paired-end format) with the inclusion of 30% PhiX control library to enhance sequence diversity (Illumina, Inc., 2018). Raw reads were demultiplexed using bcl2fastq software (version 2.20.0.422, Illumina Inc.) with default settings, permitting zero mismatches in index reads. To eliminate reads derived from the PhiX spike-in, sequences were aligned against the PhiX genome using bowtie2 (version 2.4.5, Johns Hopkins University, Baltimore, MD, USA) (Langmead and Salzberg, 2012). Reads not mapped to the PhiX reference genome were retained for further analysis. Quality control of the remaining sequences was conducted using FastQC (version 0.11.9, Babraham Bioinformatics, Babraham Institute, Cambridge, UK), which assessed base quality scores, sequence length distribution, and other metrics relevant to sequencing performance and data integrity.

#### 2.3.5 Bioinformatic analysis

Demultiplexed reads were analyzed using the QIIME2 pipeline (version qiime2-2020.8) on 16S rRNA gene sequences from microbial communities. The analysis workflow consisted of quality filtration of the sequence data and operational taxonomic unit (OTU) clustering performed using default parameter settings at 97% sequence similarity with the SILVA database (version V132). The adaptor sequences were removed, and reads with a quality score lower than 25 or length < 150 bp were discarded. The maximum number of acceptable ambiguous nucleotides was set to two, and chimeric sequences and singletons were also detected and discarded. DESeq2 (version 1.36.0; Bioconductor, University of North Carolina at Chapel Hill, Chapel Hill, NC, USA) was employed for differential abundance analysis of the gut microbiome. The laboratory with which we collaborated on bioinformatics (Koren Lab) employs the DESeq2 method for microbiome studies. We maintained the same approach and analysis as in previous works to ensure consistency in our methods. Rarified scaled OTUs were labeled by the lowest assigned taxa level possible and summarized per taxa. Differential abundance was assessed, with significant taxa determined by FDR-adjusted p-values<0.05 and |log2FoldChange| >= 0.58. For differential analysis of the different activities, sex was included as a blocking factor in the design formula. The methodological framework employed herein, along with further analytical details, is described in our previous publication by Shalmon et al (Shalmon et al., 2024), wherein the same analytical pipeline was implemented. Downstream analysis of metagenomic data was performed under the phyloseq framework (McMurdie and Holmes, 2013), including normalization of abundances and calculation of diversity metrics. Alpha-diversity indices, including the Shannon index, were computed using the phyloseq package in R.

### 2.4 Blood sampling, PBMC isolation, and microRNA profiling

#### 2.4.1 Blood sampling

Peripheral venous blood was obtained from participants via standard phlebotomy procedures, both at baseline (before exercise) and immediately after the maximal exercise stress test. Baseline measurements were derived from the resting blood samples. Blood was collected in EDTA-coated tubes (purple cap Vacutainer™, BD Biosciences, Franklin Lakes, NJ, USA) to prevent coagulation.

#### 2.4.2 Isolation of PBMCs for microRNA analysis

Peripheral blood mononuclear cells (PBMCs) were isolated using UNI-SEP Lymphocyte Separation Tubes (Novamed, Jerusalem, Israel). Whole blood was loaded into the separation tubes and centrifuged at 2700 RPM for 15 minutes to achieve stratification of PBMCs and plasma from erythrocytes. The resulting PBMC and plasma fractions were transferred to 15 mL conical tubes and centrifuged at 1700 RPM for 10 minutes. Plasma was aspirated, and the PBMC pellet was gently re-suspended in 0.5 mL of phosphate-buffered saline (PBS). The suspension was divided into two 1.5 mL microcentrifuge tubes (Eppendorf, Hamburg, Germany), centrifuged for 5 minutes at 1700 RPM, and the supernatant discarded. PBMC pellets were stored at −80 °C until further processing.

#### 2.4.3 RNA isolation

Total RNA was extracted from stored PBMC samples using TRIzol reagent (Thermo Fisher Scientific, Waltham, MA, USA), followed by phase separation with chloroform and precipitation with isopropanol. RNA quantity and purity were determined using a NanoDrop ND-1000 spectrophotometer (NanoDrop Technologies, Wilmington, DE, USA).

#### 2.4.4 Small RNA sequencing

For transcriptomic profiling, RNA libraries were prepared from PBMC RNA using the SMARTer smRNA-seq kit (Illumina, San Diego, CA, USA). Sequencing of small RNAs was performed in paired-end mode using the Illumina HiSeq platform (Illumina, San Diego, CA, USA), achieving an approximate depth of 20 million reads per sample.

#### 2.4.5 Bioinformatics and statistical analysis

Raw sequencing reads were processed using the nf-core/smrnaseq v2.24 pipeline (nf-core framework, developed by the bioinformatics community), which runs in a Nextflow environment (version 22.10.6; Seqera Labs, Barcelona, Spain). Downstream analyses were performed in R (version 4.3.1; R Core Team, Vienna, Austria) and a Linux environment. ComBat-seq (version 1.1.0; Bioconductor, Fred Hutchinson Cancer Center, Seattle, WA, USA) was applied using a negative binomial regression model to address batch-related variability across sequencing runs. DESeq2 (version 1.48.0; Bioconductor, Fred Hutchinson Cancer Center, Seattle, WA, USA) was employed for normalization and differential expression analysis, incorporating scaled covariates such as BMI and heart rate. All reported p-values were adjusted for multiple testing using the FDR correction. Empirical Bayes shrinkage (ash) adjusted log2 fold change estimates. Data visualization was conducted using the extended ggplot2 package (version 3.4.4; R Core Team, Vienna, Austria).

### 2.5 Correlation analysis between gut microbiota and PBMC microRNA expression

Building upon the processed expression data described in Section 2.4.5, a correlation analysis was performed to investigate potential interactions between the gut microbiota and host transcriptomic responses, integrating 16S rRNA gene sequencing data and small RNA transcriptomic profiles. Expression data from PBMC-derived microRNAs, processed using the nf-core/smrnaseq pipeline (version 2.2.0; nf-core framework, developed by the bioinformatics community), were batch-corrected using ComBat-seq (version 1.1.0; Bioconductor, Fred Hutchinson Cancer Center, Seattle, WA, USA) and normalized using DESeq2 (version 1.48.0; Bioconductor, Fred Hutchinson Cancer Center, Seattle, WA, USA), running on R (version 4.3.1; R Core Team, Vienna, Austria). To account for intra-individual variability and maximize statistical power, a paired experimental design was implemented by nesting subjects within activity groups, thereby adjusting for baseline differences across individuals, as described in the DESeq2 user documentation.

Functional enrichment analysis of differentially expressed microRNAs was conducted using miEAA (version 2023; Helmholtz Zentrum München, Munich, Germany), which provides pathway-level interpretation based on curated microRNA–target and disease associations.

In parallel, coexpression network analysis was conducted using the BioNERO package (version 1.4.0; Bioconductor, Fred Hutchinson Cancer Center, Seattle, WA, USA), implemented in R (version 4.3.1; R Core Team, Vienna, Austria). A signed hybrid correlation network was constructed based on Pearson correlations of the normalized count data. Network modules were identified using hierarchical clustering, and module–trait association analysis was performed using linear mixed-effects models to examine associations between expression modules and microbial taxa while accounting for relevant covariates and individual-level random effects. Descriptive statistics were used to summarize characteristics of the identified modules (e.g., size, connectivity), while quantitative associations between modules and microbial traits were assessed using inferential statistical modeling.

Given the high dimensionality of the microbiota–microRNA correlation matrix and the limited sample size, applying formal multiple testing correction would be overly conservative and substantially reduce statistical power. Similar to approaches commonly used in co-expression and network-based analyses, we therefore prioritized correlation magnitude and the distribution of nominal p-values rather than strict adjustment procedures. Specifically, only correlations with moderate-to-strong effect sizes (|r| ≥ 0.4) and a conservative nominal significance threshold (p < 0.005) were retained. Accordingly, this analysis was designed to be exploratory and hypothesis-generating, aiming to identify biologically plausible host–microbiota associations for future validation.

### 2.5.1 Assessment of potential confounding factors

To evaluate the robustness of the observed associations, each statistically significant result was further examined for potential confounding effects. Specifically, multivariable analyses were applied to assess whether the associations between gut bacterial taxa and PBMC microRNA expression remained consistent after accounting for relevant participant characteristics and clinical variables. In addition, the feature of interest was tested against other measured parameters to identify potential sources of confounding. These multivariable analyses were used for confounder assessment rather than as a primary high-dimensional modeling strategy, reflecting the exploratory design and moderate sample size of the study, and to reduce the risk of overfitting while preserving biological interpretability.

## 3 Results

### 3.1 Participant characteristics

The study groups demonstrated comparable profiles in terms of both age and body mass index (BMI). Specifically, the mean ages of the runners, cyclists, and control participants were 43.3, 45.0, and 39.4 years, respectively, while the corresponding average BMI values were 23.2, 22.9, and 23.9. These similarities indicate a relatively balanced distribution across groups, thereby reducing the likelihood of confounding influences stemming from these demographic variables. A detailed summary of participant characteristics is provided in **Table 1**.

**Table 1:** Characteristics of the participants.

Characteristic	Runners (*n* = 22)	Cyclists (*n* = 18)	Controls (*n* = 18)
Sex			
Females	9 (40.9%)	9 (50.0%)	9 (50.0%)
Males	13 (59.1%)	9 (50.0%)	9 (50.0%)
Age			
Mean (SD)	43 (6.5)	46 (7)	41 (7.4)
BMI			
Mean (SD)	23.2 (2.61)	22.9 (3.25)	23.9 (4.01)
Weekly training volume (km)			
Mean (SD)	67 (15.6)	174 (54)	5 (0)

### 3.2 Differential expression of PBMC microRNAs in response to maximal exercise in endurance athletes versus controls

When examining the impact of maximal exercise on PBMC microRNA expression in endurance athletes compared to controls, we identified nine microRNAs that were significantly upregulated or downregulated in the athletes. Five of these microRNAs showed significant changes in both runners and cyclists, while the remaining four exhibited significant changes in only one of the two athlete subgroups. Notably, these nine microRNAs were found to correlate with specific gut microbial taxa, as detailed in Section 3.4. These nine differentially expressed microRNAs were each significantly correlated with one or more specific gut bacterial taxa (FDR-adjusted p < 0.05). The direction and strength of these correlations varied, with certain microRNAs showing consistent associations across both athlete subgroups. **Table 2** presents the expression levels of these nine microRNAs (miRs) in athletes compared to controls.

**Table 2:** Differential expression of PBMC microRNAs following maximal aerobic exercise in endurance athletes compared to controls (FDR<0.05).

A. Runners vs. Controls			
microRNA		Regulation	log2FoldChange
1	hsa-miR-545-3p	Upregulated	0.26
2	hsa-miR-126-3p	Upregulated	0.99
3	hsa-miR-1292-3p	Upregulated	0.61
4	hsa-miR-6805-5p	Upregulated	0.7
5	hsa-miR-3668	Insignificant difference	
6	hsa-miR-196b-5p	Upregulated	0.2
7	hsa-miR-602	Downregulated	-0.34
8	hsa-miR-324-5p	Insignificant difference	
9	hsa-miR-365a-3p	Upregulated	1.1
B. Cyclists vs. Controls			
microRNA		Regulation	log2FoldChange
1	hsa-miR-545-3p	Upregulated	0.2
2	hsa-miR-126-3p	Upregulated	0.97
3	hsa-miR-1292-3p	Insignificant difference	
4	hsa-miR-6805-5p	Insignificant difference	
5	hsa-miR-3668	Upregulated	0.3
6	hsa-miR-196b-5p	Upregulated	0.22
7	hsa-miR-602	Downregulated	-0.51
8	hsa-miR-324-5p	Downregulated	-0.3
9	hsa-miR-365a-3p	Upregulated	0.52

**Table 2** Expression of nine PBMC microRNAs (miRs) induced by maximal exercise in endurance athletes compared to controls (FDR<0.05). The log_2_FoldChange indicates the difference in microRNA expression between groups, calculated as the logarithm base 2 of the fold change in expression levels (athletes vs. controls). A. Comparison between runners and controls. B. Comparison between cyclists and controls. The log_2_ transformation facilitates data interpretation by symmetrizing up- and downregulation and improving comparability across microRNAs.

### 3.3 Taxonomic analysis of gut microbiota composition in endurance athletes compared to controls

A taxonomic comparison of gut microbiota composition revealed seven bacterial genera with significantly higher abundance in endurance athletes—including both runners and cyclists—compared to controls. These genera were identified through differential abundance analysis using DESeq2, based on log_2_ fold change and adjusted p-values (FDR < 0.05). Specifically, the genera *Veillonella, Allisonella, Blautia, Coprococcus, Parabacteroides, Propionibacterium*, and *Butyrivibrio* were consistently enriched in the athlete group. Notably, when endurance athletes were analyzed separately as runners and cyclists, no significant differences in the abundance of these bacterial genera were observed compared to the control group. A detailed summary of the differentially abundant genera is presented in **Table 3**. These seven bacteria were found to correlate with specific PBMC microRNA expression following a maximal exercise, as detailed in Section 3.4.

**Table 3:** Differential abundance of gut microbiota in endurance athletes compared to controls (FDR < 0.05).

ID	Bacteria	log_2_FoldChange (Athletes vs. Controls)
1	*Veillonella*	2.11
2	*Allisonella*	0.24
3	*Blautia*	0.61
4	*Coprococcus*	0.31
5	*Parabacteroides*	0.34
6	*Propionibacterium*	0.81
7	*Butyrivibrio*	0.29

**Table 3** Taxonomic gut microbiota profiling in endurance athletes – including runners and cyclists - compared to controls (FDR < 0.05). log_2_FoldChange is a commonly used metric in microbiome analysis to quantify the relative difference in the abundance of specific taxa between comparison groups. A log_2_FoldChange greater than zero indicates that the taxon is relatively more abundant in endurance athletes than controls. The log_2_ transformation facilitates interpretation by symmetrizing relative differences and improving comparability across taxa.

### 3.4 Gut microbiota–PBMC microRNA correlations

Using a coexpression method on paired microRNA expression data from before and after an exercise, we found correlations with the abundance of some microbial taxa. The top few results were picked for further analysis (|r| = 0.41–0.51, *p* < 0.005).

The genus *Veillonella*, belonging to the *Veillonellaceae* family, was correlated with hsa-miR-545-3p and hsa-miR-126-3p.

Within the *Lachnospiraceae* family, the genus *Blautia* was correlated with hsa-miR-6805-5p and hsa-miR-3668, and inversely correlated with hsa-miR-6788-5p and hsa-miR-6529-3p. The genus *Coprococcus* was inversely correlated with both hsa-miR-151a-5p and hsa-miR-3196, whereas *Butyrivibrio* was directly correlated with hsa-miR-196b-5p and inversely correlated with hsa-miR-602.

In the *Tannerellaceae* family, the genus *Parabacteroides* was inversely correlated with hsa-miR-324-5p and positively correlated with hsa-miR-365a-3p. Lastly, *Propionibacterium*, a genus in the *Propionibacteriaceae* family, was positively correlated with hsa-miR-636.

**Figure 1:**
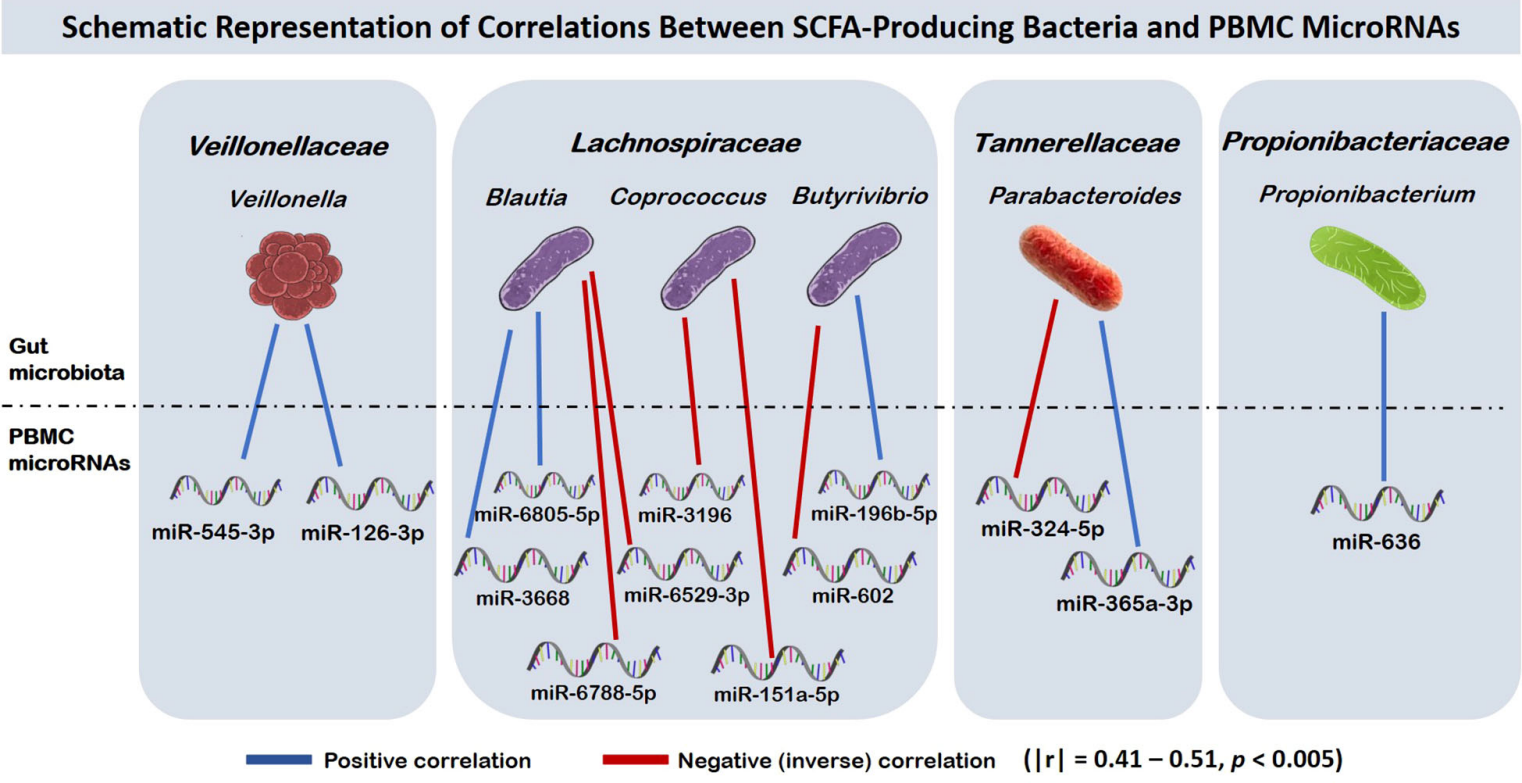
Schematic representation of correlations between SCFA-producing bacteria and PBMC microRNAs (|r| = 0.41–0.51, *p* < 0.005). The figure presents correlations observed between SCFA-producing gut bacteria (displayed at the top of the figure) and PBMC microRNA expression following exercise (displayed at the bottom). Blue lines represent positive correlations between gut bacteria and PBMC miRNAs, whereas red lines indicate negative (inverse) correlations.

13 statistically significant positive and negative (inversely correlated) associations were observed in endurance athletes, indicating potential associations between SCFA-producing gut bacteria and PBMC microRNA expression following exercise (**Figure 1**).

### 3.5 Gut microbiota diversity is correlated with the expression of a few PBMC microRNAs

A comprehensive analysis of microbial alpha-diversity metrics against metadata and PBMC microRNA expression revealed only a few strong associations. The most notable were inverse correlations between Shannon diversity and hsa-miR-8064, hsa-miR-3192-3p, and hsa-miR-12119. These were the only results that remained significant after FDR correction and also represented the top associations both before and after maximal stress exercise (P < 1×10^−4^; r < –0.5; FDR < 0.05).

## 4 Discussion

This study reveals a list of gut microbiota composition and PBMC microRNA expression following a maximal exercise test by endurance athletes, including runners and cyclists. Such correlations may indicate multilayered interactions between microbial metabolites and host gene regulatory networks, particularly in immune modulation, metabolic adaptation, and post-exercise recovery. These findings expand upon previous research that primarily focused on clinical populations by exploring microbiota–microRNA correlations in a cohort of endurance-trained athletes, offering novel insights into host–microbiome communication in the context of high-level aerobic performance.

Finding the methodology best suited for uncovering robust associations between paired microRNA expression and microbial sequencing data was a challenge in itself. We began by testing all-encompassing Omics methods, such as Sparse Canonical Correlation analysis, but found that the predictive approach might not fit the more loosely coupled data, separated both by time and sampled organ. On the other end of the spectrum, we tried a simple Pearson correlation on a per-microRNA, per-cohort-subset basis and found many correlations of varying levels of significance. We decided on a WCGNA-like approach, mostly to distill the most persistent correlations, while avoiding the spurious results of the simpler method.

We identified 13 significant correlations between gut bacterial taxa known to produce SCFAs and PBMC microRNA expression following a maximal exercise test in endurance athletes (**Figure 1**). These correlations may point to possible biological interactions between the gut microbiota and exercise-induced immune responses, although the underlying mechanisms remain to be elucidated. Specifically, they may reflect the modulation of inflammatory pathways, regulation of cytokine production, immune cell activation, and tissue repair processes—key components of physical performance, adaptation to endurance training, and recovery following strenuous exercise.

Bacteria that produce SCFAs, such as acetate, propionate, and butyrate, play a pivotal role in regulating immune cells and are considered key orchestrators of the host immune response (Ratajczak et al., 2019; Ney et al., 2023). Recent studies have demonstrated that SCFAs modulate the innate immune system, including the activation of NLRP3 inflammasomes, Toll-like receptors (TLRs), and nuclear factor-kappa B (NF-κB) signaling pathways, thereby influencing various immune cells, including PBMCs (Liu et al., 2023).

The family *Veillonellaceae* bacteria may have an adaptive advantage in athletes’ gut by lactate metabolism (lactate-utilizing bacteria), as this microbe family is more prevalent in athletes than sedentary controls in numerous human studies (Bielik et al., 2024). This anaerobic bacteria family, particularly the genus *Veillonella*, is recognized for converting exercise-induced lactate into propionate, a SCFA with known bioenergetic and mitochondrial benefits (Scheiman et al., 2019). For example, Scheiman et al. found a higher abundance of the bacterium *Veillonella atypica* among marathon runners compared to inactive people (Scheiman et al., 2019). Motiani et al. found that a significantly higher abundance of the *Veillonella* genus (including *Veillonella dispar*) was observed after moderate-intensity continuous training compared to baseline (Motiani et al., 2020). In our study, we identified positive correlations between *Veillonella* and the PBMC microRNAs hsa-miR-545-3p and hsa-miR-126-3p, as well as between the genus *Allisonella*—another member of the *Veillonellaceae* family—and hsa-miR-1292-3p. These findings are particularly noteworthy given the known biological roles of the associated microRNAs. miR-545 modulates immune cell infiltration levels and has been implicated in immunotherapeutic responses (Shen et al., 2023). miR-1292 was found to target the FZD4 gene, thereby regulating the Wnt/β-catenin pathway (Mo et al., 2024). Activating the Wnt–β-catenin signaling pathway involves many cellular functions: cell proliferation, survival, differentiation, and immune cell regulation (Haseeb et al., 2019). Furthermore, upregulation of miR-126 has been shown to enhance the angiogenic effects of exercise training via activation of the PI3K/AKT/eNOS and MAPK signaling pathways (Song et al., 2020). Taken together, these correlations may reflect a coordinated systemic adaptation whereby enhanced microbial lactate utilization fosters metabolic flexibility and contributes to shifts in miRNA expression linked to immune regulation and muscle regeneration. Specifically, *Veillonella* species may play a dual role—supporting host bioenergetics through lactate-to-propionate conversion while simultaneously modulating systemic responses via miRNA-associated signaling pathways. This dynamic host–microbe interaction could constitute a feedback mechanism essential for promoting immune resilience and facilitating tissue recovery in endurance athletes.

The genus *Blautia* has been associated with engagement in physical activity, particularly aerobic exercise, where it tends to exhibit increased abundance. For example, *Blautia* has been found to be significantly more abundant in endurance athletes compared to both sedentary controls and strength athletes (Huminska-Lisowska et al., 2024). In addition, a study involving athletes reported increased *Blautia* abundance following moderate-intensity treadmill exercise (Tabone et al., 2021). This genus possesses potential probiotic and anti-inflammatory properties, and is involved in carbohydrate metabolism, producing beneficial metabolites such as SCFAs (Liu et al., 2021; Chanda et al., 2024). In our study, a positive correlation was identified between *Blautia* and the PBMC microRNAs hsa-miR-6805-5p and hsa-miR-3668, while a negative correlation was observed with hsa-miR-6788-5p and hsa-miR-6529-3p. Although there is currently no direct research-based evidence regarding the functions of these microRNAs in immune cells, particularly under exercise conditions or in athletes, hsa-miR-6805-5p has been proposed to play a regulatory role in intracellular signaling pathways through interactions with key molecular targets. In silico analyses suggest that this microRNA may modulate the WNT signaling cascade, a pathway known for its involvement in cellular proliferation, differentiation, and tissue repair (Torso et al., 2023). Additionally, miR-6805-5p is predicted to target cell adhesion molecules such as F11R/JAM-A, which are implicated in intercellular communication and signal transduction at cell junctions, and through their potential downregulation, it may influence cytoskeletal organization, barrier integrity, and the transmission of intracellular signals (Torso et al., 2023). These regulatory actions suggest a possible role for miR-6805-5p in modulating immune cell behavior and responsiveness via control of intracellular pathways that govern activation and communication. These findings raise the hypothesis that alterations in the expression of these four PBMC microRNAs—whether upregulation or downregulation—may influence the regulation of immune responses. Nevertheless, further research is required to elucidate the biological mechanisms through which these microRNAs operate in PBMCs under exercise conditions, and to determine whether the observed correlations are causal.

*Coprococcus* is a butyrate-producing genus, with butyrate being a SCFA (Notting et al., 2023). In animal models, *Coprococcus* consistently rose with exercise (Cullen et al., 2023). Analysis of gut microbiota composition reveals that *Coprococcus* is significantly more abundant in serious runners than in sedentary healthy controls with minimal exercise habits (Duan et al., 2024). This observation aligns with other studies reporting notable differences in the prevalence of *Coprococcus* between physically active and inactive individuals, particularly highlighting its increased abundance following high-intensity endurance training (Bressa et al., 2017; Zhao et al., 2018; Moitinho-Silva et al., 2021). In our study, *Coprococcus* was positively correlated with the PBMC microRNA hsa-miR-151a-5p, while showing a negative association with hsa-miR-3196. miR-151a-5p has been implicated in a variety of biological processes (Xie et al., 2023). For example, one study proposed its role in regulating mitochondrial respiration and ATP production via modulation of cytochrome B expression (Zhou et al., 2015). In addition, as a member of the housekeeping miRNA repertoire, miR-151a-5p has shown consistent expression in both macrophages and endothelial cells under inflammatory conditions (Link et al., 2019). Similarly, overexpression of miR-3196 suppresses cell proliferation and induces apoptosis (Ji et al., 2018), suggesting that its downregulation may exert the opposite effect by promoting cell proliferation and inhibiting apoptotic pathways. In the context of peripheral blood mononuclear cells (PBMCs), such regulatory dynamics may be relevant for modulating immune cell turnover or survival in response to physiological stressors such as intense physical exercise. Although the specific role of miR-3196 in PBMCs under exercise conditions remains to be elucidated, its expression profile may contribute to shaping immune adaptation by regulating cell viability and functional responsiveness. This possibility warrants further in-depth investigation.

*Parabacteroides distasonis* has been identified in several studies as a bacterial species with a higher prevalence among athletes compared with non-athletic populations. For example, a recent study (Kulecka et al., 2023) explored differences in gut microbiome composition across various groups, including professional athletes, esports players, and students. Notably, *P. distasonis* was the only bacterial species that consistently differentiated all three study groups, with its abundance being lowest among esports players, intermediate in students, and highest in professional athletes. This gradient suggests a potential association between physical activity levels and the relative abundance of this species. *P. distasonis* has been shown to exert anti-inflammatory effects (Ma et al., 2023; Jiraskova Zakostelska et al., 2024) and to produce acetic acid, a major short-chain fatty acid (SCFA), which can subsequently be converted to methane by other microbes in complex gut communities, such as methanogens. Accordingly, fermentation by *P. distasonis* is thought to contribute indirectly to methane production (Ezeji et al., 2021). Several studies have demonstrated that methane exerts important biological effects, including anti-inflammatory and antioxidant properties, which help protect cells and organs from inflammation, oxidative stress, and apoptosis (Jia et al., 2018). In the present study, our analysis revealed a positive correlation between the genus *Parabacteroides* and PBMC-derived hsa-miR-365a-3p, and an inverse correlation with hsa-miR-324-5p. Given that *P. distasonis* is a prominent and well-characterized member of this genus that has been repeatedly associated with athletic populations, it is plausible that this species may contribute to the observed genus-level associations. Nevertheless, it should be emphasized that taxonomic classification in the current dataset is primarily interpreted at the genus level, as the sequencing approach—based on 16S rRNA gene profiling of the V4 region and 97% OTU clustering—does not reliably resolve closely related species. Upregulation of miR-365-3p has been shown to exert anti-inflammatory effects, particularly in the context of IL-17–mediated immune responses (Wang et al., 2022). This microRNA functions as a negative regulator by directly targeting ARRB2 (β-arrestin 2), a key adaptor protein involved in amplifying pro-inflammatory signaling pathways (Wang et al., 2022). Conversely, downregulation of miR-324-5p may exert immunomodulatory effects by relieving repression of its direct target, Krüppel-like factor 3 (KLF3), a transcriptional regulator involved in immune cell development, particularly within PBMC populations such as B cells and T cells. Increased KLF3 expression following miR-324-5p inhibition has been associated with suppression of pro-inflammatory gene expression and facilitation of immune resolution through apoptosis of activated immune cells (Wan et al., 2020). Taken together, these observations support the hypothesis that *Parabacteroides*-associated microbial activity may contribute to exercise-induced immune modulation in athletes by influencing the expression of specific PBMC-derived microRNAs, including hsa-miR-365a-3p and hsa-miR-324-5p. While the mechanistic pathways underlying this potential microbiota–microRNA interaction remain to be elucidated, it is plausible that microbial metabolites such as SCFAs and methane act as mediators of systemic signaling that ultimately shapes PBMC microRNA profiles in response to physical exertion. Further experimental studies are warranted to validate this proposed link and clarify its functional implications.

*Propionibacterium*, a genus of probiotic bacteria (de Assis et al., 2022) known to produce SCFAs such as propionic and acetic acid, has been identified as a strain exhibiting anti-inflammatory activity within human peripheral blood mononuclear cells (PBMCs), as supported by *in vitro* studies (Foligne et al., 2010). In this study, our analysis revealed a positive correlation between *Propionibacterium* and PBMC-derived hsa-miR-636. Overexpression of miR-636 reduces constitutive NF-κB pathway activation and then reduces inflammation (Bardin et al., 2019). Given the observed positive correlation, it is plausible to hypothesize that the presence or metabolic activity of *Propionibacterium* in the gut may contribute to the upregulation of hsa-miR-636, potentially offering a mechanistic link through which the bacterium exerts, at least in part, its anti-inflammatory effects on PBMCs.

In addition to taxon-level correlations, we also examined whether overall gut microbial diversity was associated with PBMC microRNA expression. Beyond the taxon-specific associations, exploratory analysis of microbial α-diversity revealed that only a few PBMC microRNAs exhibited robust associations with overall gut microbiota diversity. Specifically, Shannon diversity showed significant inverse correlations with hsa-miR-8064, hsa-miR-3192-3p, and hsa-miR-12119, which remained significant after FDR correction. While the current literature provides limited evidence regarding the biological roles of these specific microRNAs, none are presently recognized as established markers of inflammation. Nonetheless, their inverse relationship with microbial diversity may hold biological relevance. Higher gut microbial diversity is generally associated with metabolic and immunological homeostasis, whereas lower diversity is often linked to inflammatory or dysbiotic states (Le Chatelier et al., 2013; de Vos et al., 2022; Li et al., 2022).

Therefore, the observed pattern—where greater diversity coincides with lower expression of these microRNAs—could tentatively suggest a regulatory response consistent with a healthier or less inflammatory immune profile. However, in the absence of direct mechanistic data or previous validation of these microRNAs in immune or inflammatory contexts, this interpretation must remain speculative. It is plausible that these microRNAs reflect downstream regulatory responses related to immune cell signaling or metabolic adaptation to exercise rather than classical inflammatory pathways. Further studies employing functional assays and targeted validation are warranted to clarify whether these diversity-associated microRNAs play an active role in mediating microbiota–immune crosstalk or represent secondary markers of systemic adaptation in endurance athletes.

Most of the discussed microRNAs in this study exhibit a propensity towards anti-inflammatory activity or modulation of inflammatory responses. While the roles of several identified microRNAs in immune cells under exercise conditions remain not fully elucidated, some microRNAs, which have been demonstrated to exert anti-inflammatory effects and are associated with the suppression of pro-inflammatory gene expression, indicate a leaning towards the downregulation of inflammatory processes. Furthermore, the study’s overarching hypothesis regarding the potential of microbial interactions to foster immune resilience through miRNA-associated signaling pathways implies a bias towards the regulation, rather than the potentiation, of inflammatory states in the context of exercise.

Given that several of the correlated bacterial taxa are known producers of short-chain fatty acids (SCFAs), which have established immunomodulatory effects (Zhang et al., 2023), these findings provide preliminary support for a gut–immune signaling axis responsive to exercise.

Gut microbial metabolites provide plausible mechanistic pathways that may underpin the associations observed between SCFA-producing bacteria and PBMC microRNA regulation, particularly in the context of endurance exercise. Short-chain fatty acids (SCFAs) such as acetate, propionate, and butyrate enter the circulation and influence immune cell function through G-protein-coupled receptor (GPCR) signaling and histone deacetylase (HDAC) inhibition, thereby modulating gene expression, immune cell differentiation, and inflammatory tone (Tan et al., 2014; Koh et al., 2016). Endurance exercise is characterized by repeated transient immune activation, metabolic stress, and redistribution of circulating immune cells, conditions that may increase immune cell sensitivity to metabolite-driven signaling (Walsh et al., 2011; Campbell and Turner, 2018). Exercise can also modify SCFA availability and immune–metabolic cross-talk, potentially amplifying the effects of microbial metabolites on PBMCs (Mach and Fuster-Botella, 2017). Within this exercise-conditioned milieu, SCFA-mediated HDAC inhibition and modulation of signaling pathways relevant to both immune regulation and exercise adaptation (e.g., PI3K/AKT, mTOR, AMPK) may influence miRNA biogenesis, stability, or function in circulating immune cells (Ha and Kim, 2014; O’Neill et al., 2016). Although much of the mechanistic evidence derives from non-exercise contexts, exercise may act as a physiological amplifier rather than a distinct mechanism per se, shaping immune cell responsiveness to microbial metabolite signaling. Together, these observations provide a biologically plausible framework that is exercise-relevant and generates testable hypotheses for future functional studies in athletic populations.

A key methodological consideration in the present study is the use of baseline (resting) fecal microbiota profiles in relation to PBMC-derived microRNA responses measured following a maximal exercise bout. While this design does not allow direct assessment of acute microbiota dynamics, it is based on the premise that resting gut microbiota composition represents a relatively stable ecological and functional baseline that may shape host immune and metabolic responsiveness to physiological stressors. Several studies indicate that short-term exercise interventions often do not produce substantial changes in overall gut microbiota diversity or community structure, whereas longer-term or sustained training regimens are more likely to elicit measurable shifts in microbial composition and function (Rettedal et al., 2020; Dziewiecka et al., 2022). Importantly, however, acute exercise may transiently influence microbial metabolic activity and host exposure to microbiota-derived metabolites without detectable alterations in fecal community structure. Accordingly, the observed associations may reflect a priming effect, whereby the pre-existing gut microbial ecosystem—and its metabolic capacity, including the production and signaling of immunomodulatory metabolites such as short-chain fatty acids—modulates the magnitude or direction of exercise-induced PBMC microRNA responses (Mann et al., 2024). Such short-lived, metabolite-mediated signaling could plausibly contribute to the immediate PBMC microRNA responses observed post-exercise, even in the absence of measurable changes in fecal microbiota composition. Under this framework, resting microbiota composition may influence immune responsiveness to acute exercise rather than being substantially altered by the exercise bout itself. Nevertheless, the absence of post-exercise fecal sampling limits temporal alignment between microbiota composition and PBMC microRNA expression and precludes conclusions regarding acute microbiota changes. Future studies incorporating longitudinal microbiome sampling alongside repeated immune and transcriptomic assessments, as well as targeted metabolomic profiling, are warranted to clarify temporal relationships and underlying mechanisms.

In the present study, runners and cyclists were analyzed together as a single cohort of endurance athletes. This approach was guided by several considerations. First, both disciplines involve high aerobic demands and engage largely overlapping metabolic pathways. Second, as discussed in the previous paragraph, acute exercise bouts (~10–15 minutes) are not expected to induce markedly different microbiota responses between these endurance modalities. Third, the cohort was relatively homogeneous in terms of age, BMI, diet, and ethnicity. Finally, given the small subgroup sizes (18 cyclists and 22 runners), separate analyses would have limited statistical power and could yield unstable findings. Informal correlation analyses were performed within runners and cyclists separately to examine whether the observed miRNA–microbiota associations were consistent across both sports; no trends were observed that would be meaningful or statistically significant to report. Nevertheless, future studies with larger cohorts may explore discipline- and sex-specific patterns, providing more granular insights into host–microbiota–PBMC microRNA interactions in different endurance sports.

To the best of our knowledge, this is the first study to report significant correlations between gut microbiota composition and PBMC-derived microRNAs following exercise in endurance athletes. While these associations do not establish causality and may reflect parallel but independent processes, they nonetheless suggest potential crosstalk between the gut microbiome and host immune regulation in response to physical exertion. These preliminary findings underscore the need for further mechanistic research to elucidate the molecular pathways involved, identify microbiota-derived metabolites that may act as mediators, and ultimately determine whether a causal relationship exists between the gut microbial profile of endurance athletes and the regulation and function of their PBMCs.

Finally, several methodological considerations and cohort characteristics should be acknowledged as potential limitations. The study cohort was restricted to competitive endurance athletes of Ashkenazi Jewish descent from Israel, who followed similar omnivorous dietary patterns. While this homogeneity minimized confounding related to ethnicity and diet, it also limits the generalizability of the findings to other ethnicities, cultural backgrounds, or dietary habits. Notably, dietary patterns themselves can influence both gut microbiota composition and PBMC microRNA expression, raising the possibility that some observed correlations could be partially diet-mediated rather than solely exercise-specific. Specific dietary components, such as fiber and fermentable carbohydrates, are known to modulate gut microbiota composition and related host responses, while protein may also influence microbial populations under certain conditions. Considering these factors in future studies may help disentangle diet- versus exercise-driven effects on microbiota–miRNA interactions. Consequently, caution is warranted when extrapolating these results to broader or more heterogeneous athletic populations. Future studies including participants with diverse ethnic backgrounds and dietary patterns are needed to assess the relative contributions of diet and exercise to microbiota–miRNA interactions.

In addition, the correlation analyses should be interpreted as exploratory, as formal false discovery rate correction across all miRNA–taxa pairs was not applied. Instead, associations were identified using conservative nominal significance thresholds and effect size criteria. Accordingly, these findings should be viewed as hypothesis-generating and warrant confirmation in independent cohorts.

## 5 Conclusions

This study provides novel insights into the potential connections between gut microbiota composition and PBMC microRNA expression in response to maximal exercise in endurance athletes. Significant correlations were identified between specific SCFA–producing bacterial taxa and PBMC microRNAs implicated in immune regulation, inflammation resolution, and tissue recovery. These findings suggest that the gut microbiota may be important in modulating host immune responses and recovery processes. Specifically, microbial metabolites may influence systemic adaptations to physical exertion through microRNA-mediated signaling pathways. While causality remains to be established, these preliminary findings raise the possibility of a gut microbiota–immune axis that could contribute to athletic adaptation and resilience. Future research should investigate the mechanistic roles of these interactions and explore their potential in optimizing recovery and performance in endurance sports.

## Data Availability

The datasets presented in this study can be found in online repositories. The names of the repository/repositories and accession number(s) can be found below: https://www.ncbi.nlm.nih.gov/geo/, GSE288760.
